# Evaluating the Potential of COL8A1 as a Therapeutic Target for Chemoresistance, Disease Progression, and a Prognostic Marker in Gastric Cancer

**DOI:** 10.1111/jcmm.70621

**Published:** 2025-06-03

**Authors:** Chao Xu, MuZhen He, HongYuan Chen, LiangJie Chi, XiangYu Wang, ShuYuan Li, QingShui Wang, Yao Lin, FangQin Xue

**Affiliations:** ^1^ Department of Gastrointestinal Surgery Shengli Clinical Medical College of Fujian Medical University, Fujian Provincial Hospital, Fuzhou University Affiliated Provincial Hospital Fuzhou China; ^2^ Department of Radiology Shengli Clinical Medical College of Fujian Medical University, Fujian Provincial Hospital, Fuzhou University Affiliated Provincial Hospital Fuzhou China; ^3^ Fujian‐Macao Science and Technology Cooperation Base of Traditional Chinese Medicine‐Oriented Chronic Disease Prevention and Treatment, Innovation and Transformation Center Fujian University of Traditional Chinese Medicine Fuzhou China

**Keywords:** chemotherapy, COL8A1, gastric cancer, immunotherapy, recurrence

## Abstract

This study aimed to identify key genes associated with post‐chemotherapy recurrence in gastric cancer patients. Gene expression data from multiple cohorts were analysed to determine differentially expressed genes between recurrent and non‐recurrent cases. A prognostic risk model incorporating COL8A1, HSPB7 and SLIT2 was developed and validated across six independent cohorts. The risk score demonstrated significant associations with disease‐free and overall survival, tumour grade and molecular subtypes. Notably, the risk score showed potential as a predictor of immunotherapy response, outperforming established markers such as microsatellite instability score and Epstein–Barr virus status. Analysis of the tumour immune microenvironment revealed a correlation between risk score and M2 macrophage infiltration. A nomogram integrating the risk score with clinical factors demonstrated high accuracy in predicting patient survival. Further investigation of COL8A1 revealed its significant role in gastric cancer cell proliferation, metastasis, and chemoresistance. In vitro and in vivo experiments showed that COL8A1 knockdown inhibited cancer cell growth, invasion, and metastasis while enhancing chemosensitivity. These findings provide valuable insights into the molecular mechanisms of gastric cancer recurrence and offer potential biomarkers for prognosis and treatment response prediction. The study highlights the importance of integrating genomic data with clinical information to improve patient stratification and personalised treatment strategies in gastric cancer management.

## 1 Introduction

Gastric cancer remains a significant contributor to cancer‐associated mortality worldwide [1, 2, 3]. While surgery is the primary therapeutic modality for advanced stages of gastric cancer, its efficacy is limited by the high incidence of metastasis. Consequently, relying solely on surgical interventions for managing gastric cancer is suboptimal, with numerous cases of disease recurrence post‐treatment. Mounting evidence suggests that adjuvant chemotherapy can improve survival outcomes in patients with advanced gastric cancer following curative surgery [4, 5, 6]. Agents such as 5‐fluorouracil (5‐FU), platinum‐based compounds, anthracyclines, taxanes and irinotecan have demonstrated efficacy in managing advanced disease stages [7]. However, the benefits of adjuvant chemotherapy are not universal across all patients.

5‐FU, in particular, stands as a cornerstone in adjuvant treatment regimens. Key enzymes involved in 5‐FU metabolism, including thymidylate synthase (TS), thymidine phosphorylase (TP) and dihydropyrimidine dehydrogenase (DPD), have been proposed as predictive markers of 5‐FU efficacy [8, 9, 10]. However, their predictive validity remains contentious. Consequently, there is an urgent need for additional molecular markers to accurately predict clinical outcomes and guide patient‐specific chemotherapy. Understanding the molecular underpinnings of recurrence is crucial for improving prognoses and stimulating innovative therapeutic developments. Recent advancements in genomic profiling have provided remarkable insights into the intricate biological mechanisms driving the aggressiveness and treatment resistance of gastric cancer.

COL8A1, a key component of type VIII collagen, plays a critical role in extracellular matrix remodelling and tumour microenvironment dynamics. Previous studies have demonstrated its involvement in tumour progression and metastasis in various cancers, including breast cancer and non‐small cell lung cancer (NSCLC). In breast cancer, COL8A1 facilitates growth through activation of the FAK/Src signalling pathway [11]. Similarly, it promotes NSCLC progression via EGFR activation mediated by IFIT1/IFIT3 [12]. Emerging evidence indicates that COL8A1 expression is associated with poor prognosis and chemoresistance, underscoring its potential as both a prognostic marker and a therapeutic target. However, studies investigating COL8A1 in gastric cancer remain limited, particularly regarding its role in recurrence and treatment resistance.

In this study, we leveraged genomic data repositories to identify differentially expressed genes (DEGs) associated with recurrence in gastric cancer patients treated with chemotherapy. Among the identified genes, COL8A1 emerged as a critical player, showing a strong correlation with recurrence and poor prognosis. We developed and validated a risk score model incorporating COL8A1 to enhance recurrence prediction and improve personalised treatment strategies. These findings aim to bridge the gap in understanding the molecular mechanisms underlying gastric cancer recurrence and provide novel insights into its clinical management.

## 2 Methods

### 2.1 Data Acquisition

Publicly available gene expression datasets (GSE13861 [13], GSE26899 [14], GSE26253 [15], GSE66229 [14] and GSE26901 [14]) were obtained from the GEO database. The Affy package in R was employed to normalise and preprocess the microarray data using the Robust multi‐array average method. GC gene expression data, quantified as fragments per kilobase million (FPKM), along with corresponding clinical information, were retrieved from TCGA via the UCSC XENA platform [16]. These FPKM values were subsequently converted to TPM for downstream analysis. Additionally, patient cohorts for PD‐L1 treatment in gastric cancer (KIM cohort) and melanoma (Hugo cohort) were accessed through the tumour immune dysfunction and exclusion (TIDE) database.

### 2.2 Identification of Overlapping Genes and Survival Analysis

To identify consistently up‐regulated genes across all three cohorts, we constructed Venn diagrams. Subsequently, we employed Kaplan–Meier survival analysis and Cox univariate survival analysis to elucidate the relationship between gene expression and patient prognosis. This approach enabled us to pinpoint genes associated with poor survival outcomes.

### 2.3 Prognostic Risk Model Development

We developed a prognostic gene signature using the least absolute shrinkage and selection operator (LASSO) Cox regression model. This method was implemented using the glmnet package in R, enabling us to identify the most predictive genes for inclusion in the risk score calculation. For each patient, we computed a risk score based on a linear combination of gene expression levels, with each gene weighted by its corresponding LASSO coefficient.

### 2.4 Model Validation

We validated the risk score model using additional independent cohorts. The effectiveness of the model was assessed through Kaplan–Meier survival curves, ROC curves and AUC analyses. To further demonstrate its prognostic significance, we visually depicted the survival status and expression patterns of the genes incorporated in the model.

### 2.5 Immune Microenvironment Analysis

The CIBERSORT algorithm was employed to estimate the relative proportions of various immune cell types within tumour samples based on their gene expression signatures. We evaluated the relationship between the risk scores and immune cell infiltration, along with their correlation to the expression of immune checkpoints.

### 2.6 Patients and Specimens

A total of 10 gastric cancer tissue samples were collected from Fujian Provincial Hospital between April 2019 and September 2020. The samples included five from patients with recurrent gastric cancer and five from patients without recurrence. All patients underwent chemotherapy treatment with a regimen of oxaliplatin, tegafur, and apatinib. Patients were classified as having recurrent gastric cancer if the disease recurred within three years post‐surgery, and as non‐recurrent if no recurrence was observed within the same period. This investigation was conducted in accordance with the protocols and guidelines approved by the Fujian Provincial Hospital's Research Ethics Committee. Written informed consent was obtained from all participants under a protocol sanctioned by the institution.

### 2.7 Immunohistochemistry (IHC) Staining Analysis

We performed IHC to assess the expression profile of COL8A1 in gastric cancer specimens using a standard immunoperoxidase staining technique. Tissue sections were incubated with an anti‐COL8A1 primary antibody (Catalogue: ab198899, Abcam) at a 1:100 dilution. After staining, two pathologists independently quantified COL8A1 expression using a composite scoring system. The extent of cellular positivity was divided into four categorical tiers: Category 1 (0%–25%), Category 2 (26%–50%), Category 3 (51%–75%) and Category 4 (76%–100%). In parallel, the intensity of the staining was graded on a scale from 0 to 3, where 0 represented no detectable staining, 1 indicated weak staining, 2 denoted moderate staining and 3 reflected strong staining. A final weighted score for each sample was derived by multiplying the percentage of positively stained cells by the corresponding intensity grade.

### 2.8 Cell Culture

AGS and SNU‐1 were obtained from the ATCC. These cells were propagated in DMEM enriched with 10% foetal bovine serum and 1% penicillin–streptomycin antibiotic, maintained in a humidified atmosphere of 5% CO_2_ at 37°C.

### 2.9 CCK‐8 Proliferation Assay

To evaluate cell proliferation, AGS and SNU‐1 cells were plated in 96‐well plates at a density of 2 × 10^4^ cells per well and cultured for 24, 48 and 72 h. At 4 h before the conclusion of each time point, 10‐μL of CCK‐8 solution was introduced into each well. Following a further 2‐h incubation period, the absorbance was measured at 450 nm using a microplate reader.

### 2.10 Invasion Assay

For the invasion assay, AGS and SNU‐1 cells were placed on inserts within an invasion chamber using a serum‐free medium. The lower compartment contained DMEM with 10% FBS to incentivise cell movement. Following a 48‐h incubation, the invasive cells were fixed with methanol and stained using crystal violet. The cells that did not invade were carefully wiped off from the top side of the insert membrane. Invasion was quantified by counting cells in six randomly chosen fields per membrane.

### 2.11 Migration Assay

Upon reaching near‐confluence, AGS and SNU‐1 cell monolayers were subjected to scratch wounds. Cells were washed with phosphate‐buffered saline (PBS) to clear debris and received serum‐free medium to exclude growth factor influence on cell migration. After 48 h of incubation, images were captured to determine wound closure.

### 2.12 Zebrafish Xenograft Assay

The zebrafish xenograft model was established to evaluate the in vivo tumour growth and metastatic potential of AGS colorectal cancer cells. Zebrafish ( *Danio rerio* ) were obtained from Fuzhou Bio‐Service Biotechnology Co. Ltd. (Fuzhou, China) and maintained under standard laboratory conditions at 28.5°C with a 14:10‐h light–dark cycle. For xenotransplantation, AGS cells were first digested with trypsin and labelled with 5 μM 1,1′‐dioctadecyl‐3,3,3′,3′‐tetramethylindocarbocyanine perchlorate (Dil; Meilun Biotechnology, China), a red fluorescent lipophilic membrane dye, to enable precise tracking of cell migration and proliferation. The xenotransplantation procedure was performed using GB100T‐8P glass capillaries and FemtoJet 4i microinjectors (Eppendorf, Germany). Approximately 200 labelled AGS cells were carefully injected into the central yolk sac or ventral yolk cavity of zebrafish larvae aged 2–3 days post‐fertilisation, with each experimental group consisting of ten larvae to ensure statistical reliability. Fluorescence microscopy was used to monitor tumour cell dynamics at two critical time points: for proliferation assessment, imaging was conducted at 2 and 48 h post‐transplantation (hpt) to evaluate initial cell distribution and tumour cell growth, respectively; for metastasis assessment, tail fluorescence was recorded at 2 and 24 h post‐transplantation (hpt) to analyse cell migration and metastatic potential. Notably, experimentation on zebrafish larvae younger than 5 days old does not require ethics committee approval [17, 18]. Our study adhered to ARRIVE guidelines for reporting animal research.

### 2.13 Chemosensitivity Assay

We utilised the MTT assay to evaluate the cytotoxic effects of 5‐FU (Sigma‐Aldrich) on AGS and SNU‐1 cells. Cells were exposed to various concentrations of 5‐FU to assess dose‐dependent cytotoxicity. To ensure data reliability and reproducibility, we conducted three independent experiments. The IC50 values, representing the concentration of 5‐FU required to inhibit cell viability by 50%, were calculated using GraphPad Prism 8 software. This approach allowed us to quantitatively assess the sensitivity of AGS and SNU‐1 cells to 5‐FU treatment.

### 2.14 RNA Extraction and Quantitative PCR

Total RNA was isolated from AGS and SNU‐1 cells using TRIzol reagent. cDNA was synthesised using a reverse transcription kit, followed by quantitative PCR amplification with gene‐specific primers. For COL8A1, we used the following primer sequences: forward 5′‐GCTGCTGGGAATACTGTTCA‐3′ and reverse 5′‐GGGAGGTATGGGTACTCTTT‐3′. GAPDH served as the reference gene, with primer sequences: forward 5′‐GCGGGGCTCTCCAGAACATCAT‐3′ and reverse 5′‐CCAGCCCCAGCGTCAAAGGTG‐3′. Relative gene expression was quantified using the 2^−ΔΔ*Ct*^ method. All qPCR reactions were performed in triplicate to ensure reproducibility.

### 2.15 Statistical Analysis

Data analysis was conducted using Prism 6.0 software. The results are expressed as the mean ± SD from three independent experiments. Differences between means were evaluated using one‐way ANOVA or Student's *t*‐test. To evaluate survival differences between high‐ and low‐risk patient groups, a log‐rank test was utilised. The independence of the risk score was assessed through multiple Cox regression analyses.

## 3 Results

### 3.1 Workflow of This Study

In our study, we identified a total of 155 gastric cancer patients across three datasets: 49 from GSE13861, 67 from GSE26899 and 39 from GSE26901, all of whom had undergone chemotherapy. We conducted an analysis of DEGs between patients who experienced recurrence and those who remained recurrence‐free following chemotherapy. Based on these findings, we developed a prognostic model. The comprehensive workflow of our study is illustrated in Figure S1.

### 3.2 Identification of Key Genes Associated With Post‐Chemotherapy Recurrence in GC Patients

**FIGURE 1:**
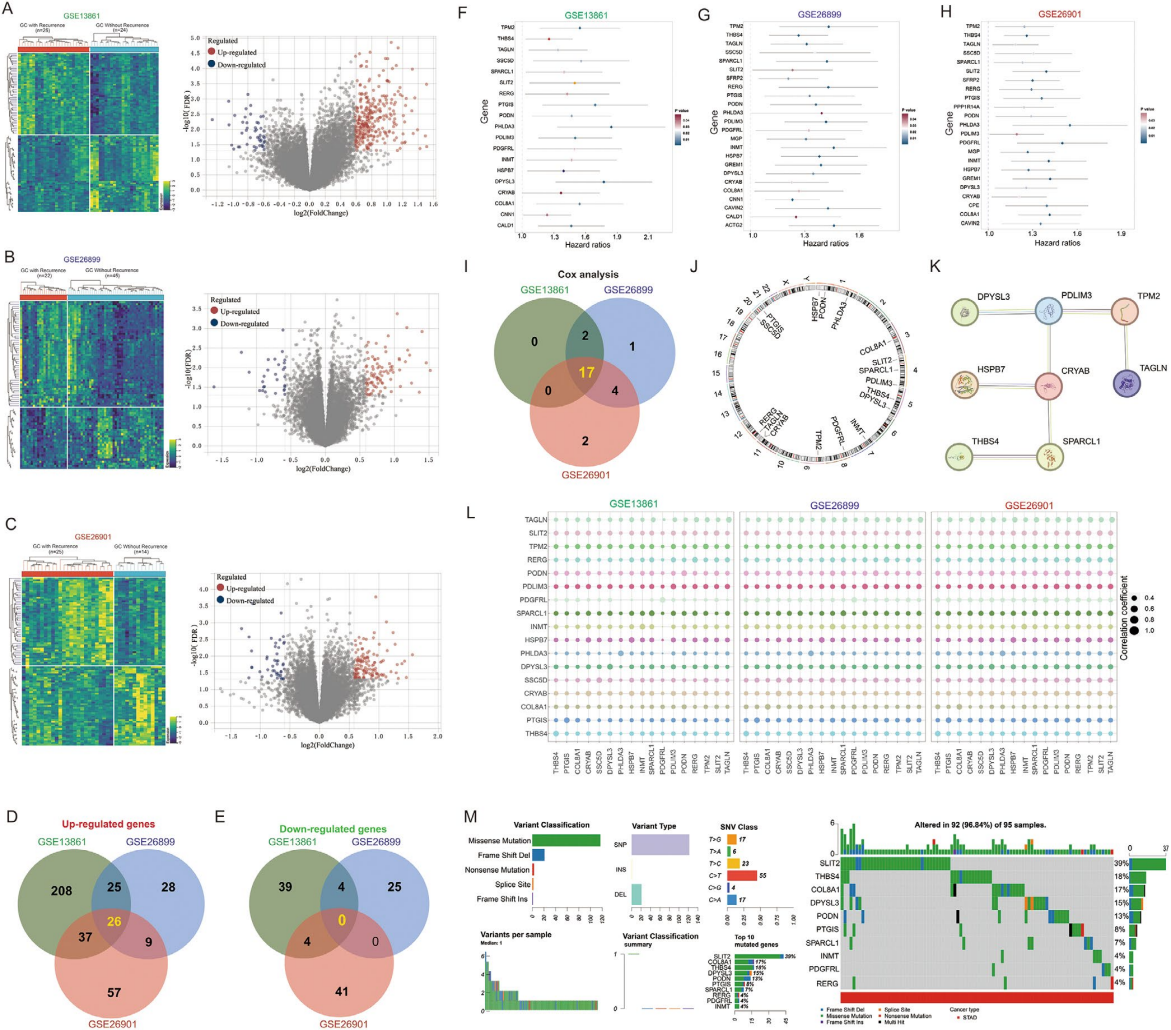
Identification of key genes for recurrence after chemotherapy in GC. (A–C) Volcano plots and heatmaps display DEGs in GC patients post‐chemotherapy recurrence versus non‐recurrence in datasets (A) GSE13861, (B) GSE26899 and (C) GSE26901; (D, E) Venn diagrams show the intersection of (D) up‐regulated and (E) down‐regulated genes across three datasets; (F–H) Univariate Cox analysis of the 26 intersecting genes for prognosis in (F) GSE13861, (G) GSE26899 and (H) GSE26901 GC patient datasets; (I) Venn diagram displays the intersection of 17 genes with prognostic value across three datasets; (J) Distribution of 17 genes on chromosomes; (K) Protein–protein interaction analysis network of the 17 genes; (L) Expression correlation analysis of the 17 genes; (M) Mutation status of the 17 genes in GC tissues.

The primary objective of this study was to identify crucial genes associated with GC recurrence following chemotherapy. We analysed data from the GSE13861, GSE26899 and GSE26901 cohorts, all of which included patients who had received chemotherapy. Differential gene expression analysis unveiled significant alterations in gene expression patterns among patients with recurrent disease across all three cohorts. In the GSE13861 cohort, we observed a marked up‐regulation of 296 genes and down‐regulation of 47 genes in patients who experienced recurrence (Figure 1A). The GSE26899 cohort exhibited 88 up‐regulated and 29 down‐regulated genes in recurrent cases (Figure 1B), while the GSE26901 cohort demonstrated 125 up‐regulated and 45 down‐regulated genes (Figure 1C). Venn diagram analysis revealed a core set of 26 genes consistently up‐regulated across all three datasets in patients with recurrent disease compared to those without recurrence (Figure 1D). Notably, we found no genes consistently down‐regulated across all three cohorts (Figure 1E). The 26 up‐regulated genes include: *PPP1R14A*, *PDGFRL*, *RERG*, *SDPR*, *GREM1*, *PTGIS*, *INMT*, *PHLDA3*, *CRYAB*, *HSPB7*, *SLIT2*, *CPE*, *ACTG2*, *MGP*, *DPYSL3*, *PDLIM3*, *SPARCL1*, *TPM2*, *PODN*, *COL8A1*, *SFRP2*, *TAGLN*, *CNN1*, *CCL1* and *THBS4*.

A subsequent Cox univariate survival analysis revealed the following findings: 19 genes were associated with poor prognosis in the GSE13861 cohort, 24 such genes were identified in the GSE26899 cohort, and 23 in the GSE26901 cohort (Figure 1F–H). Further Venn diagram analysis highlighted 17 common genes that were correlated with adverse prognosis across all datasets studied (Figure 1I). Notably, all these identified genes are located on autosomal chromosomes (Figure 1J). A protein–protein interaction network analysis demonstrated connections among eight of these genes: DPYSL3, PDLIM3, TPM2, TAGLN, CRYAB, HSPB7, THBS4 and SPARCL1 (Figure 1K). Moreover, an examination of expression correlations among the 17 identified genes showed a strong interdependence (Figure 1L). Mutational analysis indicated that SLIT2 had the highest mutation frequency, followed by THBS4, COL8A1, DPYSL3, PODN, PTGIS, SPARCL1, INMT, PDGFRL and RERG (Figure 1M).

### 3.3 Prognostic Model Construction for Gastric Cancer Patients Based on Recurrent Key Genes

**FIGURE 2:**
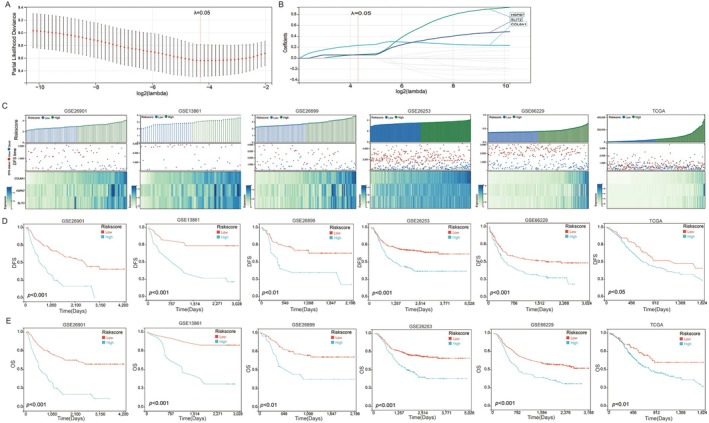
Prognostic model construction based on recurrence key genes for GC patients. (A, B) LASSO Cox regression to construct a prognostic model for GC patients; (C) Risk score, survival status and expression levels of the three genes across six GC datasets; The impact of risk score on patients' (D) DFS and (E) OS in six GC datasets. The grouping of risk score‐low and risk score‐high is established based on the optimal threshold. Statistical analysis was performed using the log‐rank test to assess differences in survival outcomes.

We subsequently focused our analysis on the 17 genes identified from the intersecting sets. A LASSO Cox regression analysis was conducted on the GSE26901 cohort to calculate regression coefficients, with the model demonstrating optimal performance when incorporating three genes (Figure 2A). The prognostic model was derived from these coefficients (Figure 2B) as follows: Risk score = (0.233 × COL8A1 expression) + (0.066 × HSPB7 expression) + (0.060 × SLIT2 expression). We present the survival statuses, risk scores and expression levels of these three genes for patients from the GSE26901 (training) cohort and five validation cohorts: GSE13861, GSE26899, GSE26253, GSE66229 and TCGA (Figure 2C). Based on their risk scores, we stratified gastric cancer patients into high‐ and low‐risk subgroups. Kaplan–Meier analysis revealed that patients with low‐risk scores had significantly longer DFS times compared to those with high‐risk scores across all six gastric cancer cohorts (Figure 2D). Moreover, patients with low‐risk scores demonstrated significantly longer overall survival times than their high‐risk counterparts (Figure 2E).

**FIGURE 3:**
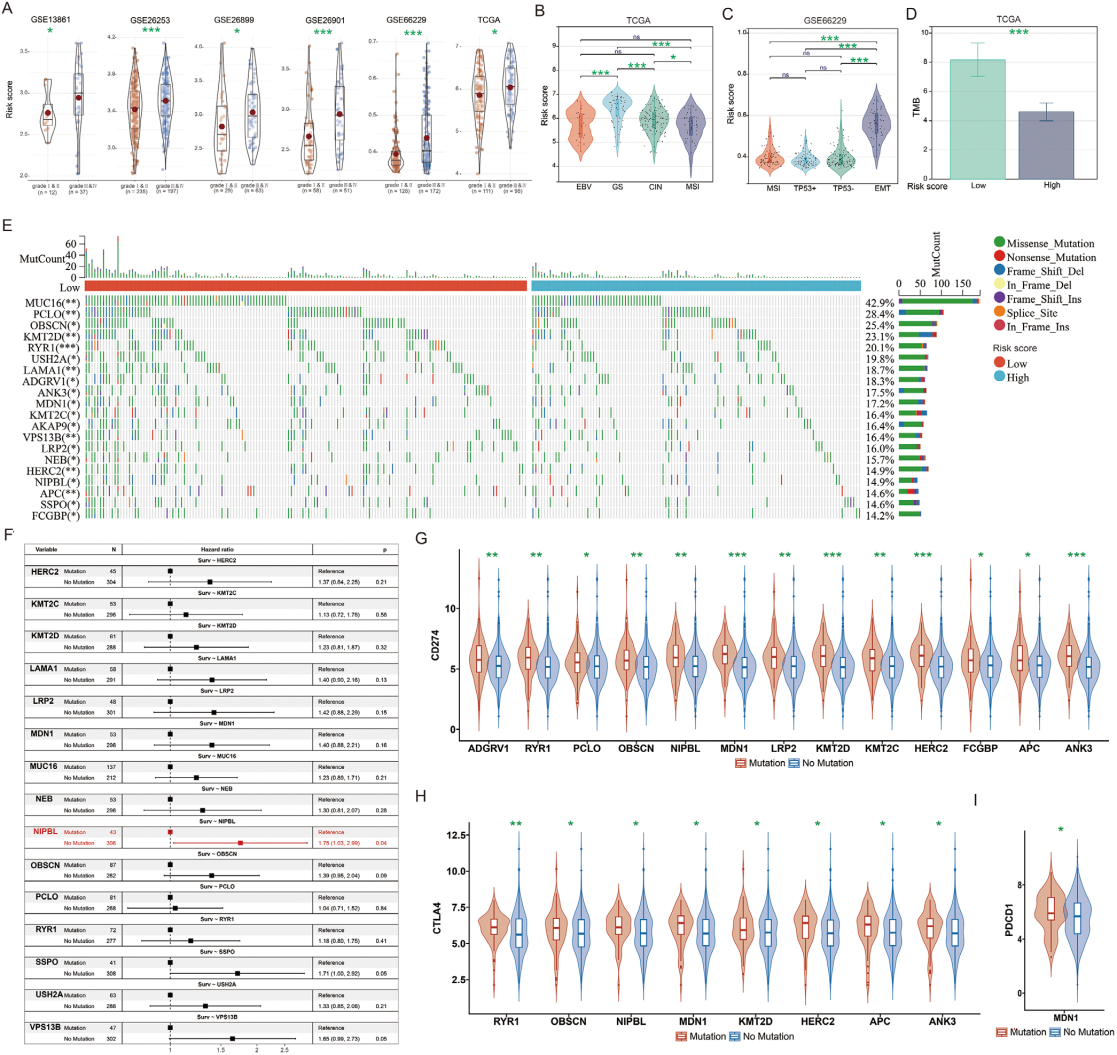
Correlation analysis of risk score with clinical characteristics of GC. (A) Difference analysis of risk scores across different grades in six GC datasets; (B) Different expression analysis of risk scores among subtypes in the TCGA dataset; (C) Different expression analysis of risk scores among subtypes in the GSE66229 dataset; (D) Differential expression analysis of TMB between high‐risk and low‐risk score subgroups; (E) Gene mutation analysis in high‐risk and low‐risk score subgroups; (F) Impact of differentially mutated genes on the prognosis of patients with GC in high‐risk and low‐risk score subgroups; Correlation analysis of mutated genes with (G) CD274, (H) CTLA4, and (I) PDCD1 expression. *p < 0.05; **p < 0.01; ***p < 0.001.

We further investigated the relationship between risk scores and clinical features of gastric cancer. Our findings revealed significantly higher risk scores for grade III & IV gastric cancers compared to grade I & II across six cohorts: GSE26901, GSE13861, GSE26899, GSE26253, GSE66229 and TCGA (Figure 3A), indicating a clear association between risk score and gastric cancer progression. In the TCGA and GSE66229 datasets, gastric cancers were classified into four molecular subtypes. Within the TCGA cohort, the Epstein–Barr virus (EBV) and microsatellite instability (MSI) subtypes exhibited the lowest risk scores (Figure 3B). Conversely, in the GSE66229 cohort, the EMT subtype demonstrated the highest risk score (Figure 3C).

Given that TMB is a potential biomarker for immunotherapy due to its role in neoantigen production, we examined TMB variations between subtypes. Our analysis revealed elevated TMB levels in the Low‐risk score subtype compared to the High‐risk score subtype (Figure 3D). Further analysis of genomic alterations between low‐ and high‐risk score subtypes showed generally higher mutation frequencies in genes associated with the low‐risk score subtype (Figure 3E). Notably, gastric cancer patients with NIPBL mutations demonstrated significantly improved prognosis compared to those without such mutations (Figure 3F). Considering that elevated immune checkpoint expression may indicate better immunotherapy response, we explored the relationship between mutated genes and immune checkpoint expression. Our analysis revealed significantly higher levels of the immune checkpoint CD274 in patients carrying mutations in ADGRV1, RYR1, PCLO, OBSCN, NIPBL, MDN1, LRP2, KMT2D, KMT2C, HERC2, FCGBP, APC and ANK3 compared to those with wild‐type variants (Figure 3G). Patients with mutations in RYR1, OBSCN, NIPBL, MDN1, KMT2D, HERC2, APC and ANK3 exhibited notably higher CTLA4 immune checkpoint expression (Figure 3H), while MDN1 mutant patients showed increased expression of the PDCD1 immune checkpoint compared to wild‐type patients (Figure 3I).

### 3.4 Risk Score as a Predictor for Immunotherapy Response in Gastric Cancer

**FIGURE 4:**
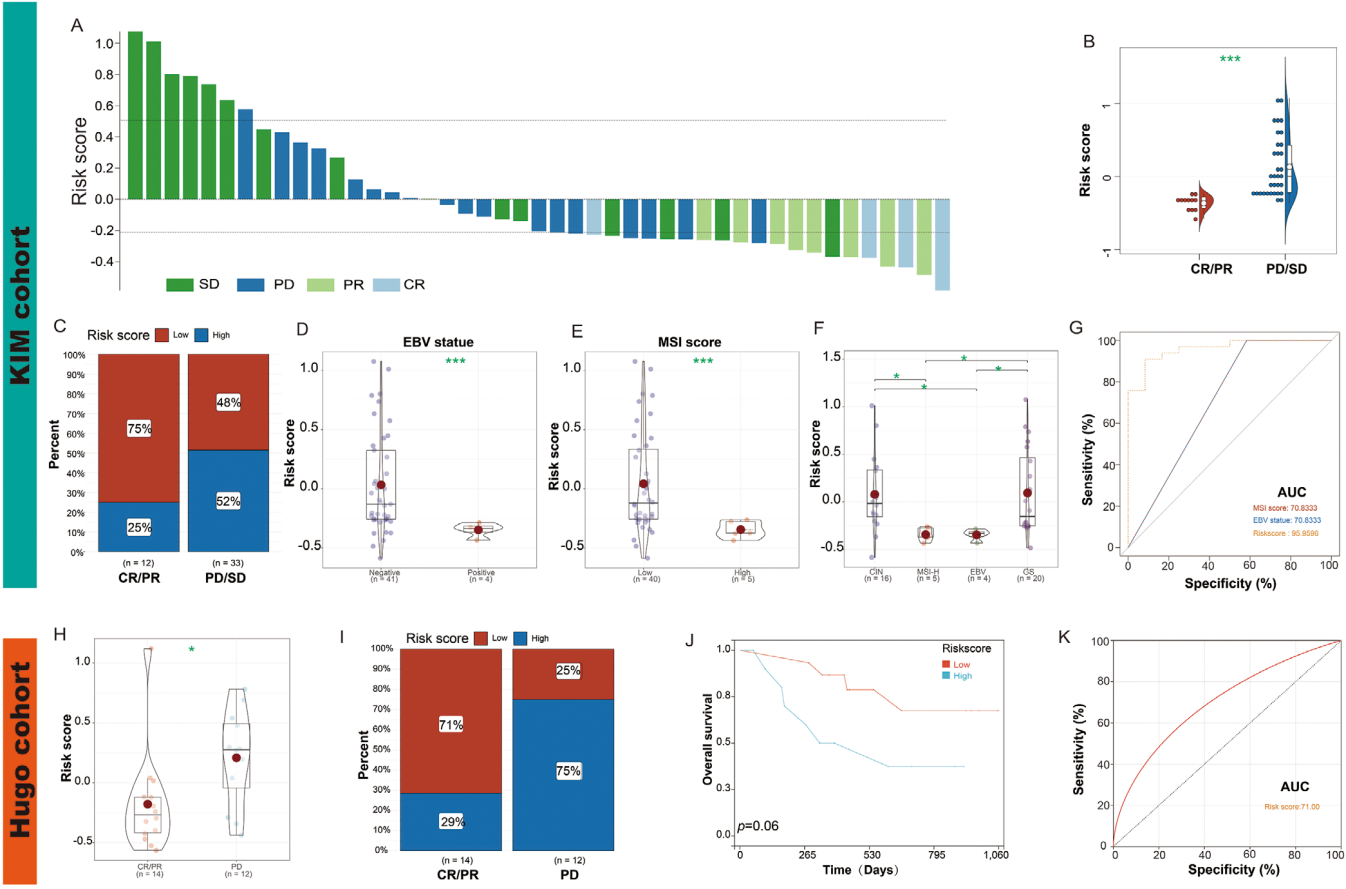
Prediction of immunotherapy response in GC by risk score. (A) Correlation between risk score and immunotherapy response in the KIM cohort; (B) Difference analysis of risk scores between PR/CR group and PD/SD group; (C) Proportions of patients with different responses to immunotherapy among two risk score subtypes; (D) Difference in risk score between EBV‐negative and ‐positive statuses; (E) Difference in risk score between low MSI score and high MSI score groups; (F) Different expression analysis of risk score among different subtypes in the KIM cohort; (G) Predictive value of risk score, MSI status, and EBV status in patients receiving immunotherapy in different cohorts; (H) Difference analysis of risk score between PR/CR group and PD group in the Hugo cohort; (I) Proportions of patients with different immunotherapy responses among two risk score subtypes in the Hugo cohort; (J) Impact of risk score on the prognosis of gastric cancer patients in the Hugo cohort; (K) Predictive value of risk score in the Hugo cohort. The grouping of Risk score‐Low and Risk score‐High is established based on the optimal threshold. Statistical analysis was performed using the log‐rank test to assess differences in survival outcomes.*p < 0.05; ***p < 0.001.

Given the promising outcomes of immunotherapy, particularly immune checkpoint inhibitors such as PD‐1 and PD‐L1, in various malignancies including gastric cancer, we further explored the predictive potential of the risk score in the KIM and Hugo cohorts. We examined the association between risk score and immunotherapeutic response in the KIM cohort, which comprised patients with advanced gastric cancer undergoing PD‐L1 blockade treatment. Risk score distribution varied among patients with different treatment responses (Figure 4A). Notably, risk scores were significantly elevated in patients categorised as having disease progression (PD) or stable disease (SD), compared to those achieving partial response (PR) or complete response (CR) (Figure 4B). Interestingly, the PR/CR group predominantly consisted of individuals with low‐risk scores (75%), while high‐risk scores were more prevalent in the PD/SD group (52%) (Figure 4C). These findings suggest that a high‐risk score may serve as a potential negative predictor of immunotherapy response in gastric cancer patients. Moreover, considering that MSI score and EBV status are primary indicators of immunotherapy efficacy, we observed that patients with low MSI scores or negative EBV status exhibited higher risk scores compared to those with high MSI scores or positive EBV status (Figure 4D,E). The KIM cohort classifies gastric cancer into four molecular subtypes based on distinct molecular characteristics. Our analysis revealed that within this cohort, the EBV‐positive and MSI‐H subtypes demonstrated the lowest risk scores (Figure 4F). Furthermore, upon calculating the AUC for the risk score, we found its value (0.96) surpassed those of both the MSI score and EBV status (Figure 4G), underscoring its potential as a robust predictor of immunotherapy response.

Analysis of the Hugo cohort, comprising melanoma patients undergoing PD‐1 blockade therapy, yielded results consistent with our previous findings. Notably, individuals classified as responders exhibited significantly lower risk scores compared to non‐responders (Figure 4H). A majority of patients experiencing PR or CR had low risk scores (71%), whereas high risk scores were predominantly observed in the PD cohort (75%) (Figure 4I). Survival analysis revealed that patients with low‐risk scores demonstrated prolonged survival times compared to their high‐risk counterparts. However, due to the limited sample size, these survival differences did not reach statistical significance (Figure 4J). ROC curve analysis yielded an AUC of 0.71 for the risk score, underscoring its robust potential as a predictive marker for immunotherapy outcomes across various malignancies.

### 3.5 The Correlation Between Risk Score and the Immune Microenvironment in GC

To assess the relationship between the immune microenvironment and risk score, we employed CIBERSORT to analyse the enrichment scores of immune‐related cells. Across all six cohorts, our analysis consistently revealed a significantly higher proportion of Macrophage M2 cells in the high‐risk score subgroup compared to the low‐risk score subgroup (Figure S2). This was the only immune cell type to show such a consistent pattern.

**FIGURE 5:**
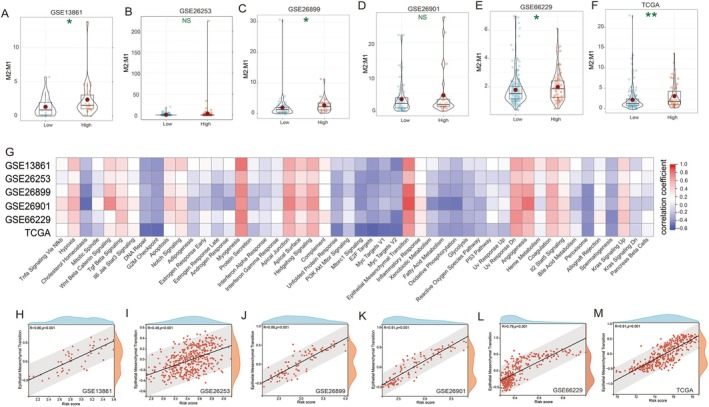
Relationship between risk score and GC immune microenvironment. (A) Ratio of M2/M1 in datasets GSE13861, (B) GSE26253, (C) GSE26899, (D) GSE26901, (E) GSE66229, (F) TCGA dataset; (G) Association between risk score and signalling pathways in six datasets; (H) Correlation between risk score and EMT in datasets GSE13861, (I) GSE26253, (J) GSE26899, (K) GSE26901, (L) GSE66229, (M) TCGA dataset. NS, *p* > 0.05; *p < 0.05; **p < 0.01.

Furthermore, we examined the ratio of M2 to M1 macrophages in all six cohorts. Our findings demonstrated that the M2/M1 ratio was consistently elevated in the high‐risk score subgroup (Figure 5A–F), reinforcing the association between risk score and macrophage polarisation.

To elucidate the underlying biological mechanisms, we conducted GSVA to identify differentially activated signalling pathways between the subgroups. Notably, the EMT signalling pathway exhibited a strong correlation with the risk score across all six datasets (Figure 5G–M). This consistent association suggests a potential mechanistic link between our risk score model and EMT, a process known to play a crucial role in cancer progression and metastasis.

### 3.6 Nomogram Survival Model Establishment

To determine whether the risk score could serve as an independent prognostic factor, we conducted univariate and multivariate Cox regression analyses. The univariate Cox regression analysis identified the risk score as a significant risk factor (HR = 3.52, 95% CI: 1.93–6.42) (Figure S3A). Importantly, even after adjusting for potential confounding factors, the multivariate analysis confirmed that the risk score remained an independent prognostic indicator for GC patients (HR = 2.35, 95% CI: 1.22–4.52) (Figure S3B).

We developed a nomogram model using multivariate Cox and stepwise regression analyses to predict 1‐, 3‐ and 5‐year survival rates, incorporating age, stage, and risk score as key variables (Figure S3C). The model's accuracy in estimating these survival rates was validated through calibration curves (Figure S3D). ROC analysis demonstrated that the nomogram exhibited high predictive accuracy for 1‐, 3‐ and 5‐year survival in GC patients (Figure S3E).

Furthermore, DCA revealed that the nomogram model provided greater net benefits compared to any other predictor used in this study (Figure S3E). Notably, when patients were stratified based on their nomogram scores, substantial differences in survival were observed among various subgroups (Figure S3G).

### 3.7 High COL8A1 Expression Is an Unfavourable Prognostic Factor for GC Patients

**FIGURE 6:**
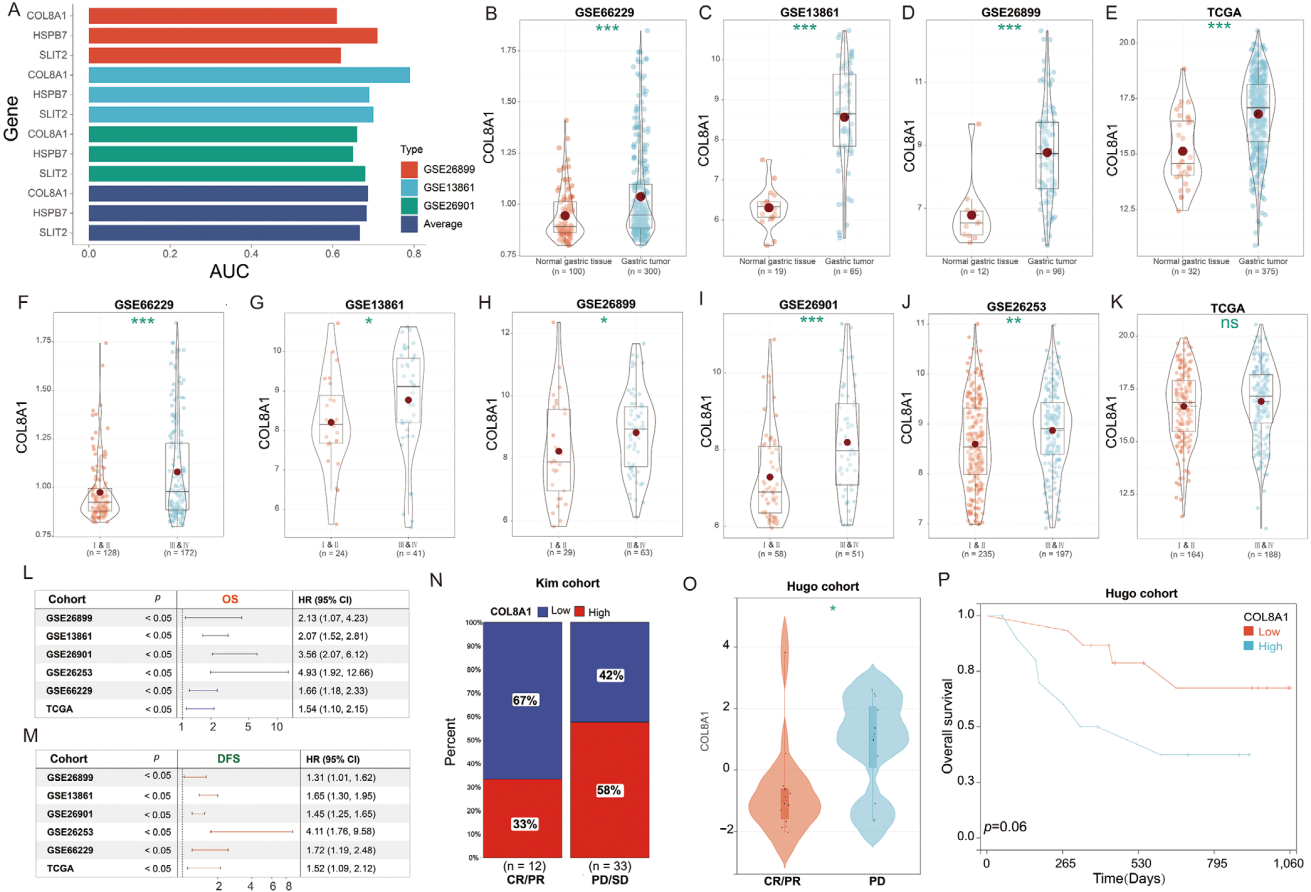
High COL8A1 expression is an unfavourable prognostic factor for GC patients. (A) ROC analysis of COL8A1 in predicting recurrence after chemotherapy for diagnosed GC; (B) Differential expression of COL8A1 in cancer versus adjacent non‐cancerous tissues in datasets GSE66229, (C) GSE13861, (D) GSE26899, (E) TCGA; (F) Differential expression of COL8A1 in different grades in datasets GSE66229, (G) GSE13861, (H) GSE26899, (I) GSE26901, (J) GSE26253, (K) TCGA; (L) OS survival analysis of COL8A1 in six datasets; (M) DFS survival analysis of COL8A1 in six datasets; (N) Proportion of patients with different responses to immunotherapy against COL8A1; (O) Differential expression analysis of COL8A1 between PR/CR group and PD group in Hugo cohort; (P) Survival analysis of COL8A1 in Hugo cohort. The grouping of COL8A1‐Low and COL8A1‐High is established based on the optimal threshold. Statistical analysis was performed using the log‐rank test to assess differences in survival outcomes. NS, *p* > 0.05; *p < 0.05; **, *p* < 0.01; ***p < 0.001.

We subsequently evaluated the efficacy of three genes in diagnosing GC recurrence in post‐chemotherapy patients. ROC analysis revealed that COL8A1 consistently demonstrated the highest average AUC value across all three cohorts (Figure 6A). We analysed the differential expression of COL8A1 between GC tissues and adjacent non‐tumorous tissues using the GSE66229, GSE13861, GSE26899 and TCGA cohorts. Our findings consistently showed significantly elevated COL8A1 expression in GC tissues compared to adjacent non‐tumorous tissues (Figure 6B–E).

Across six GC cohorts (GSE26901, GSE13861, GSE26899, GSE26253, GSE66229 and TCGA), COL8A1 expression was notably higher in grades III and IV compared to grades I and II (Figure 6F–K). Survival analysis demonstrated that COL8A1 expression was closely associated with both OS and DFS in GC patients across all six datasets (Figure 6L,M).

Utilising the KIM cohort, we investigated the relationship between COL8A1 and immunotherapy response in GC. Our analysis revealed that low COL8A1 expression was predominant in the PR/CR group (67%), whereas high COL8A1 expression was primarily observed in the progressive PD/SD group (58%) (Figure 6N). Analysis of the Hugo cohort further corroborated these findings, showing significantly elevated COL8A1 expression in the PD group compared to the PR/CR group. Moreover, elevated COL8A1 expression emerged as a negative prognostic indicator for immunotherapy outcomes in GC patients (Figure 6O,P).

### 3.8 COL8A1 Promotes Proliferation, Metastatic Potential and Drug Resistance of GC Cells

**FIGURE 7:**
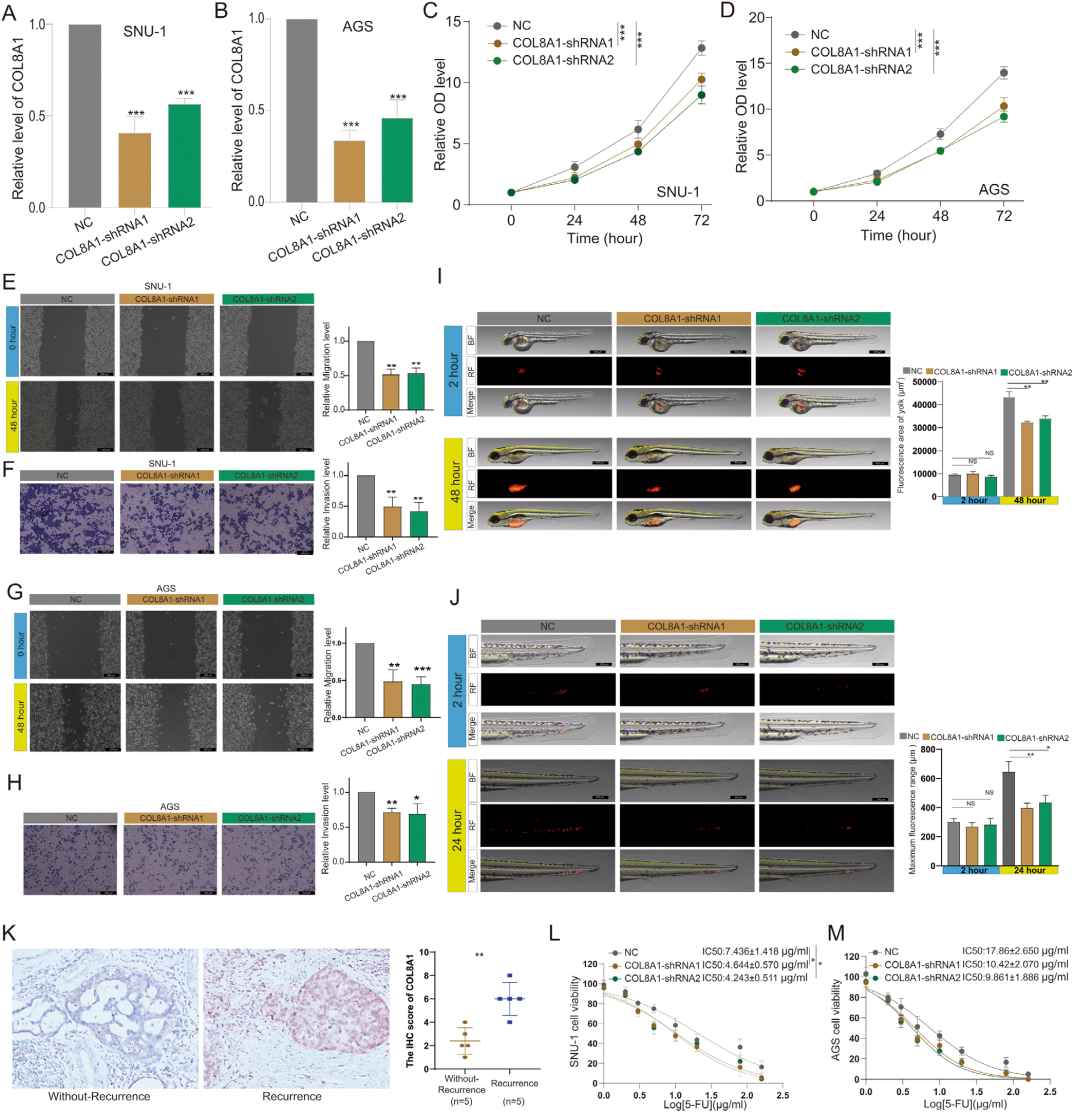
COL8A1 promotes proliferation, metastatic potential and drug resistance of GC cells. (A) RT‐PCR detection of COL8A1 expression after knockdown in (A) SNU‐1 and (B) AGS cells; CCK8 assay for changes in proliferation ability after COL8A1 knockdown in (C) SNU‐1 and (D) AGS cells; Effect of COL8A1 knockdown on migration and invasion abilities of SNU‐1 cells (E) migration and (F) invasion; Effect of COL8A1 knockdown on migration and invasion abilities of AGS cells (G) migration and (H) invasion; Effect of COL8A1 knockdown on in vivo proliferation and metastasis capabilities of AGS cells in zebrafish (I) proliferation and (J) metastasis; (K) Immunohistochemical detection of COL8A1 expression in GC tissues. IC50 assay for changes in drug IC50 of 5‐FU after COL8A1 knockdown in (L) SNU‐1 and (M) AGS cells. NS, *p* > 0.05; **, *p* < 0.01; ***, *p* < 0.001.

To evaluate the impact of COL8A1 on the functional capabilities of GC cells, we performed COL8A1 knockdown in SNU‐1 and AGS cell lines (Figure 7A,B). CCK8 assay results demonstrated that COL8A1 knockdown significantly suppressed the proliferation of both SNU‐1 and AGS cells (Figure 7C,D). Further investigations using scratch and invasion assays revealed that COL8A1 knockdown significantly impaired the migration and invasion capabilities of both cell lines (Figure 7E–H). These findings suggest a crucial role for COL8A1 in promoting GC cell aggressiveness.

To validate our in vitro findings in an in vivo context, we established a zebrafish xenograft model of AGS cells to assess tumour proliferation and metastasis. At 2 h post‐transplantation (hpt), there was no significant difference in fluorescence intensity between the COL8A1 knockdown group (COL8A1‐shRNA) and the negative control group (NC), indicating similar initial tumour cell distribution. However, by 48 h post‐transplantation, the fluorescence intensity in the COL8A1 knockdown group was significantly reduced compared to the NC group (Figure 7I), demonstrating that COL8A1 knockdown effectively inhibited tumour cell proliferation within the zebrafish model. In addition to proliferation, we also analysed the metastatic potential of AGS cells using tail fluorescence imaging (Figure 7J). At 2 h post‐transplantation, there was no significant difference in tail fluorescence area between the COL8A1 knockdown group and the NC group. However, by 24 h, the COL8A1 knockdown group exhibited a significant reduction in tail fluorescence area compared to the NC group, suggesting that the knockdown of COL8A1 effectively curtailed tumour cell migration. This in vivo evidence corroborates our in vitro results and underscores the potential of COL8A1 as a therapeutic target for inhibiting gastric cancer progression.

Lastly, we examined COL8A1 expression in clinical samples and its impact on chemoresistance. Immunohistochemical analysis was performed on tissue samples from 5 cases of non‐recurrent gastric cancer and 5 cases of recurrent gastric cancer. The results revealed a significant up‐regulation of COL8A1 expression in recurrent gastric cancer tissues compared to non‐recurrent cases (Figure 7K).

Furthermore, we investigated the influence of COL8A1 on the chemosensitivity of GC cells to 5‐FU. Our findings demonstrated that knockdown of COL8A1 substantially reduced the half‐maximal inhibitory concentration (IC50) of 5‐FU in both SNU‐1 and AGS cell lines (Figure 7L,M). This observation suggests that COL8A1 may play a role in modulating chemoresistance in GC cells.

## 4 Discussion

The field of oncology continually grapples with the complex challenges of treatment resistance and cancer recurrence, particularly in gastric cancer, which remains a leading cause of cancer‐related mortality worldwide [19, 20, 21, 22]. Identifying specific genes associated with gastric cancer recurrence post‐chemotherapy is crucial for advancing precision medicine approaches. This groundbreaking research provides a foundation for tailoring treatment strategies based on individual patients' genetic profiles. By elucidating genetic markers indicative of higher recurrence risk, this study not only aids in prognosis but also offers valuable insights into the molecular mechanisms underlying chemoresistance [23]. Consequently, this work has the potential to facilitate the development of novel therapeutic targets and improve patient management, potentially impacting survival rates and quality of life for those affected by this aggressive malignancy.

Our comprehensive analysis of gene expression profiles has yielded significant insights into the genes associated with recurrence and poor prognosis in post‐chemotherapy gastric cancer patients. The identification of 26 consistently up‐regulated genes across three independent cohorts (GSE13861, GSE26899 and GSE26901) highlights the potential mechanistic role these genes may play in cancer recurrence. Notably, we developed a prognostic model incorporating COL8A1, HSPB7 and SLIT2, which demonstrated satisfactory performance, showing promise for future clinical applications. Subsequent survival analyses reinforced the model's validity, revealing a significant correlation between gene‐based risk scores and both disease‐free and overall survival across multiple cohorts.

COL8A1, initially identified as a primary component of type VIII collagen in corneal and vascular endothelial cells [24], has recently been implicated in the progression of various cancers. Studies have shown that COL8A1 promotes triple‐negative breast cancer growth through FAK/Src signalling pathway activation [11] and facilitates NSCLC progression by influencing EGFR activation, mediated by IFIT1/IFIT3 [12]. HSPB7 has been found ineffective in inhibiting amorphous aggregation of model proteins but highly efficient in preventing aggregation of glutamine‐rich huntingtin fragments [25, 26]. Emerging evidence suggests HSPB7 plays a significant role in tumour development [27, 28, 29]. SLIT2, a member of the SLIT family of secreted glycoproteins and the human homologue of Drosophila Slit2 protein [30], acts as a ligand for the ROBO1 receptor, facilitating intracellular signal transduction, including GTPase‐activating proteins [31, 32]. Research has shown that tumour‐induced activation of the TLR3‐SLIT2 signalling pathway in endothelial cells enhances metastasis [33]. In gastric cancer specifically, SLIT2 promotes metastasis by activating the kinase NEK9 [34].

Recent studies have established a strong correlation between genetic mutations and tumour development [35]. TMB, which quantifies somatic mutations in coding regions of the genome [36], is hypothesised to increase neoantigen production in severely mutated tumours, leading to T cell infiltration. Our results demonstrate that low‐risk score subtypes exhibit higher somatic mutation rates and TMB, while high‐risk score subtypes show the opposite trend. Furthermore, multiple studies indicate that gastric cancer patients with MSI and EBV subtypes respond more effectively to PD1 inhibitors like pembrolizumab [37]. Our study found significantly lower risk scores for EBV and MSI subtypes compared to other subtypes, suggesting the risk score as a reliable model for stratifying gastric cancer patients.

Our research also demonstrates the risk score's efficacy as a biomarker for predicting outcomes in gastric cancer patients receiving PD‐L1 blocking immunotherapy. Patient classification into low‐risk and high‐risk groups correlated closely with treatment response, with high‐risk scores associated with disease progression or stability, and low‐risk scores predominating among patients achieving partial or complete remission. These findings are particularly relevant given the increasing adoption of immunotherapy in cancer treatment. While immune checkpoint inhibitors targeting PD‐1 and PD‐L1 have revolutionised cancer treatment, predicting patient response remains challenging [38]. Our risk score showed superior predictive power compared to MSI and EBV status, indicating its potential to guide clinical decision‐making and personalised treatment strategies.

Our findings suggest that the established risk score model holds significant promise for guiding personalised therapeutic strategies in gastric cancer. By integrating gene expression data with clinical parameters, clinicians could categorise patients into distinct risk profiles, thereby aiding in the selection of optimal treatment regimens. High‐risk patients, for instance, may benefit from more aggressive or combination therapies, whereas low‐risk individuals might be spared overtreatment and associated toxicities. Moreover, this model can be integrated into existing predictive tools to refine surgical and adjuvant decision‐making, ultimately moving towards a more tailored and patient‐centric approach. Future prospective clinical trials are warranted to validate the efficacy and safety of this risk stratification model and to assess its impact on long‐term patient outcomes.

The TME significantly impacts tumour progression, treatment response and clinical outcomes. Our study highlights the role of the immune microenvironment, particularly the ratio of M2‐like to M1‐like macrophages, which positively correlates with risk scores. M0 macrophages can polarise into M1 and M2 phenotypes, with M1 macrophages mediating pro‐inflammatory responses and M2 macrophages facilitating anti‐inflammatory processes [39]. Previous studies suggest that a higher presence of M1 macrophages correlates with better cancer outcomes, while increased M2 macrophages often associate with poorer prognoses [40, 41].

Our nomogram survival model, incorporating the risk score, presents a potentially powerful tool for clinicians. Its high accuracy, evidenced by AUC values and calibration curves, suggests robust individualised prognosis prediction. Decision curve analysis further validates the clinical utility of our nomogram, demonstrating superior net benefit compared to standard predictors.

The strong association of COL8A1 with various aspects of gastric cancer pathogenesis reinforces its potential as a therapeutic target. Our experimental data show that silencing COL8A1 attenuates cell proliferation and invasiveness while enhancing sensitivity to chemotherapeutic agents like 5‐FU. These in vitro findings, supported by in vivo validation using zebrafish models, suggest a multifaceted role for COL8A1 in the malignant cascade.

While our findings are promising, several limitations warrant consideration. The retrospective nature and use of pre‐existing database cohorts may introduce selection bias and limit result extrapolation. Additionally, our in vivo work was limited to zebrafish models, which, while useful for certain aspects of tumour biology, may not fully capture the complexity of the human tumour microenvironment. Future studies focusing on diverse genetic backgrounds and leveraging patient‐derived xenografts or organoids may provide additional depth to our understanding.

## Author Contributions

**Chao Xu:** conceptualization (equal), data curation (equal), writing – original draft (equal). **MuZhen He:** formal analysis (equal), investigation (equal), methodology (equal). **HongYuan Chen:** software (equal), validation (equal). **LiangJie Chi:** investigation (equal), methodology (equal). **XiangYu Wang:** resources (equal), software (equal). **ShuYuan Li:** data curation (equal), formal analysis (equal), resources (equal). **QingShui Wang:** conceptualization (equal), data curation (equal), writing – original draft (equal). **Yao Lin:** writing – original draft (equal), writing – review and editing (equal). **FangQin Xue:** writing – original draft (equal), writing – review and editing (equal).

## Disclosure

Animal Studies: Experimentation on zebrafish larvae younger than 5 days old does not require ethics committee approval. Our study adhered to ARRIVE guidelines for reporting animal research.

## Ethics Statement

The study was conducted in accordance with the guidelines and regulations approved by the Research Ethics Committee of the Fujian Provincial Hospital.

## Consent

Written informed consent was obtained from all patients participating in the study under an institutionally approved protocol.

## Conflicts of Interest

The authors declare no conflicts of interest.

## Supporting information


Figures S1–S3.

## Data Availability

The datasets used to support the conclusion of this study were collected from publicly available databases including TCGA (https://portal.gdc.cancer.gov/) and GEO database (https://www.ncbi.nlm.nih.gov/geo/).
