# Supplementary material for: Evaluating the Potential of COL8A1 as a Therapeutic Target for Chemoresistance, Disease Progression, and a Prognostic Marker in Gastric Cancer
Source: J Cell Mol Med. 2025 Jun 3;29(11):e70621. doi: 10.1111/jcmm.70621 (PMC12133449; doi:10.1111/jcmm.70621)
Supplement: Supplementary file 1 — Figures S1–S3. [file JCMM-29-e70621-s001.docx]

**
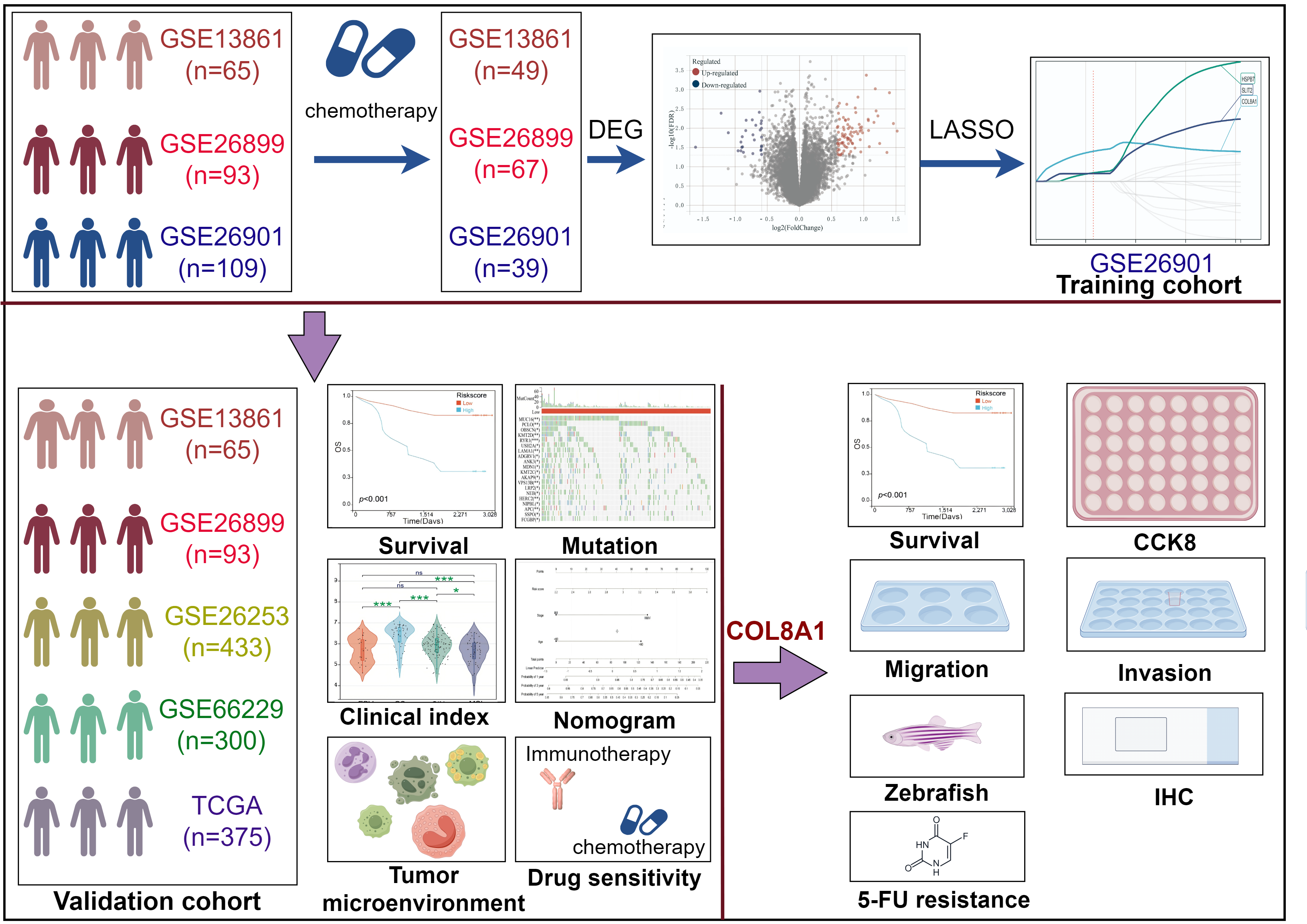
Supplementary Figure 1. Flowchart for comprehensive analysis of prognostic model based on chemotherapy recurrence-related genes in gastric cancer**


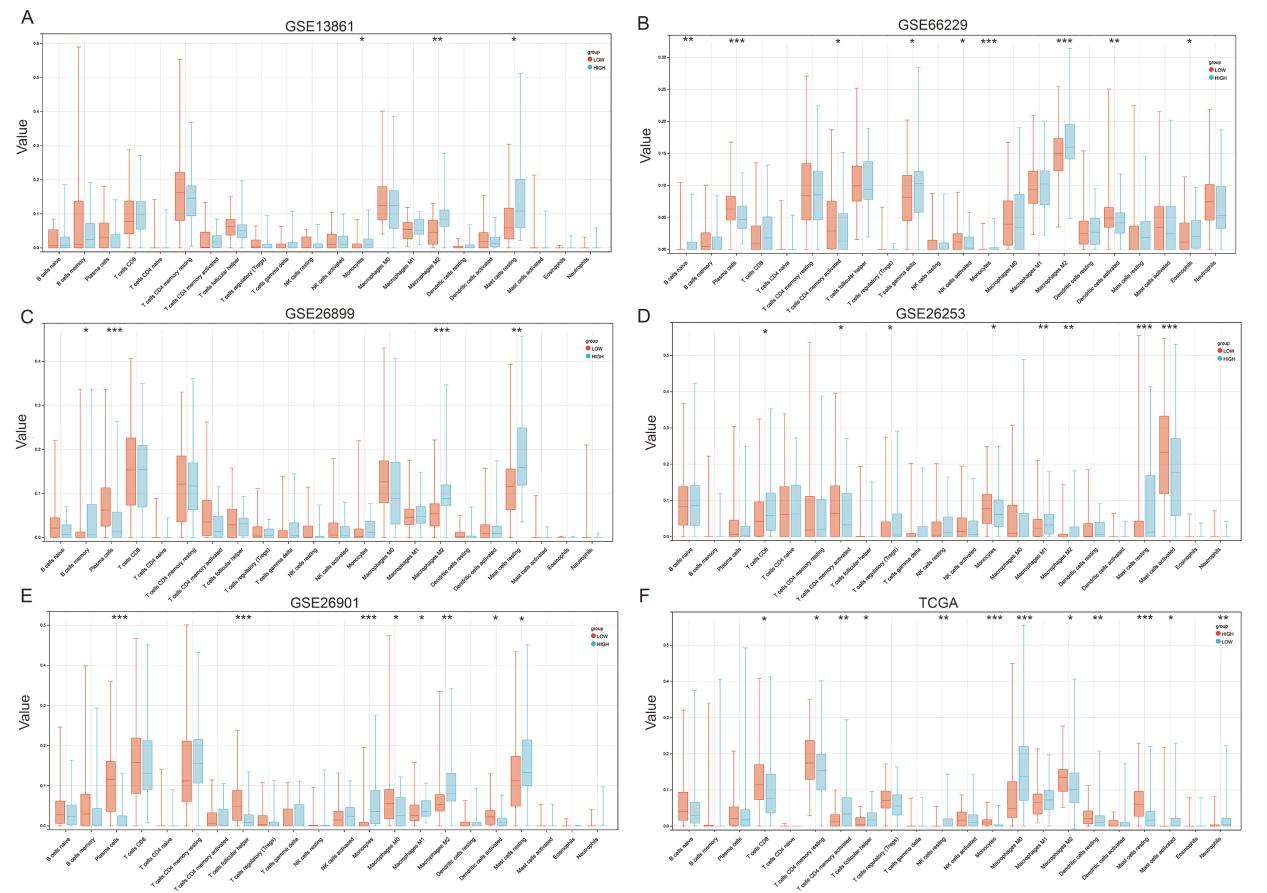
**Supplementary Figure 2. Correlation analysis of risk score with Immune-related cells in gastric cancer tissues**

Correlation analysis of risk score with immune-related cells in datasets (a) GSE13861, (b) GSE66229, (d) GSE26899, (d) GSE26253, (e) GSE26901, and (f) TCGA. *, *p*<0.05; **, *p*<0.01; ***, *p*<0.001.


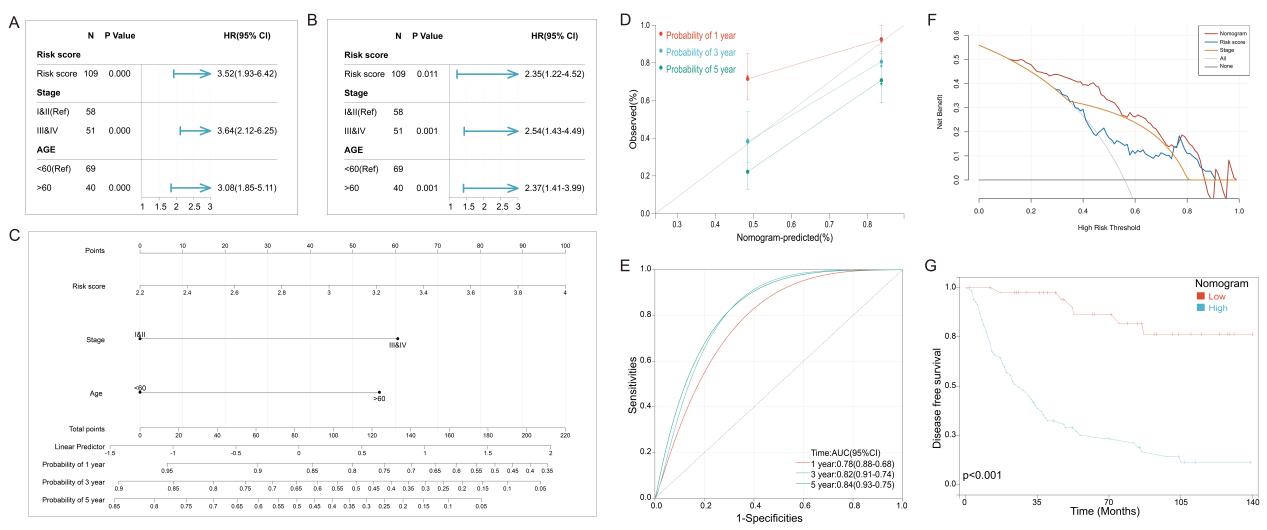


**Figure 3. Establishment and evaluation of the Cox proportional Hazards survival model**

(A) Univariate analysis of clinicopathological features and risk score in GSE26901 cohort; (B) Multivariate analysis of clinicopathological features and risk score in GSE26901 cohort; (V) Establishing a nomogram to predict prognosis of gastric cancer patients; (D) Calibration plot showing the probability of 1-year, 3-year, and 5-year survival rates in GSE26901 cohort; (E) ROC analysis of nomogram in GSE26901 cohort; (F) DCA for total survival rate prediction by nomogram; (G) Kaplan-Meier analysis based on nomogram scoring for two GC groups.
